# QuitSMART Utah: an implementation study protocol for a cluster-randomized, multi-level Sequential Multiple Assignment Randomized Trial to increase Reach and Impact of tobacco cessation treatment in Community Health Centers

**DOI:** 10.1186/s13012-020-0967-2

**Published:** 2020-01-30

**Authors:** Maria E. Fernandez, Chelsey R. Schlechter, Guilherme Del Fiol, Bryan Gibson, Kensaku Kawamoto, Tracey Siaperas, Alan Pruhs, Tom Greene, Inbal Nahum-Shani, Sandra Schulthies, Marci Nelson, Claudia Bohner, Heidi Kramer, Damian Borbolla, Sharon Austin, Charlene Weir, Timothy W. Walker, Cho Y. Lam, David W. Wetter

**Affiliations:** 1 Center for Health Promotion and Prevention Research, Department of Health Promotion & Behavioral Sciences, University of Texas Health Science Center at Houston School of Public Health, 7000 Fannin St, Houston, TX 77030 USA; 2 Center for Health Outcomes and Population Equity, Huntsman Cancer Institute, University of Utah, 2000 Circle of Hope Dr, Salt Lake City, UT 84112 USA; 3 Department of Biomedical Informatics, University of Utah, 421 Wakara Way #140, Salt Lake City, UT 84108 USA; 4 Association for Utah Community Health, 860 E 4500 S, Murray, UT 84107 USA; 5 Department of Population Health Sciences, University of Utah, 295 Chipeta Way, Salt Lake City, UT 84108 USA; 6 Institute for Social Research, University of Michigan, 426 Thompson St, Ann Arbor, MI 48104 USA; 7 Utah Department of Health, 288 N 1460 W, Salt Lake City, UT 84116 USA

**Keywords:** Tobacco cessation, Adaptive intervention, Implementation science, Health information technology, Quitline, Implementation strategy

## Abstract

**Background:**

Tobacco use remains the leading cause of death and disability in the USA and is disproportionately concentrated among low socioeconomic status (SES) populations. Community Health Centers (CHCs) are a key venue for reaching low SES populations with evidence-based tobacco cessation treatment such as Quitlines. Electronic health record (EHR)-based interventions at the point-of-care, text messaging (TM), and phone counseling have the potential to increase Quitline reach and are feasible to implement within CHCs. However, there is a lack of data to inform how, when, and in what combination these strategies should be implemented. The aims of this cluster-randomized trial are to evaluate multi-level implementation strategies to increase the Reach (i.e., proportion of tobacco-using patients who enroll in the Quitline) and Impact (i.e., Reach × Efficacy [efficacy is defined as the proportion of tobacco-using patients who enroll in Quitline treatment that successfully quit]) and to evaluate characteristics of healthcare system, providers, and patients that may influence tobacco-use outcomes.

**Methods:**

This study is a multilevel, three-phase, Sequential Multiple Assignment Randomized Trial (SMART), conducted in CHCs (*N* = 33 clinics; *N* = 6000 patients). In the first phase, clinics will be randomized to two different EHR conditions. The second and third phases are patient-level randomizations based on prior treatment response. Patients who enroll in the Quitline receive no further interventions. In phase two, patients who are non-responders (i.e., patients who do not enroll in Quitline) will be randomized to receive either TM or continued-EHR. In phase three, patients in the TM condition who are non-responders will be randomized to receive either continued-TM or TM + phone coaching.

**Discussion:**

This project will evaluate scalable, multi-level interventions to directly address strategic national priorities for reducing tobacco use and related disparities by increasing the Reach and Impact of evidence-based tobacco cessation interventions in low SES populations.

**Trial registration:**

This trial was registered at ClinicalTrials.gov (NCT03900767) on April 4th, 2019.

Contribution to literature

- QuitSMART Utah is a Sequential Multiple Assignment Randomized Trial (SMART) to optimize the sequencing and integration of effective, scalable, multi-level implementation strategies for evidence-based tobacco cessation treatment.
- This pragmatic trial is conducted in real-world healthcare settings that serve socioeconomically disadvantaged populations (Community Health Centers).
- Researchers and practitioners alike struggle to apply guidance from implementation science in the development and evaluation of implementation efforts. The study presents a conceptual framework to guide the use of both behavioral science theory and implementation frameworks in the development and evaluation of implementation strategies at multiple levels.

## Background

Tobacco use is the leading cause of preventable death and disability in the United States (US) and 30% of all cancers are directly attributable to tobacco use [1]. Over the last several decades, tobacco use has become increasingly concentrated among individuals living in poverty, with low education, and the uninsured and unemployed [2]. Consequently, the implementation and dissemination of “evidence-based tobacco control strategies” was identified as a priority recommendation for reducing health disparities by the National Institutes of Health [3].

Despite conclusive evidence that evidence-based tobacco cessation treatment is both effective and cost-effective, it is grossly underutilized [4–9]. Although 70% of current tobacco users want to quit and over 50% attempt to quit each year [10], over 90% of those quit attempts fail, in large part because the majority (80%) fail to utilize any form of evidence-based treatment [7, 11, 12]. Low SES tobacco users have less awareness and access to evidence-based cessation treatments [13, 14], are less likely to participate in treatment, and have even less success in quitting [13–18].

### Evidence-based tobacco cessation: Quitlines

One of the most significant advances in tobacco control over the last several decades has been the creation of tobacco cessation Quitlines [7]. Quitlines provide both pharmacotherapy and behavioral interventions via phone counseling, online platforms, and text messaging. The efficacy of Quitlines and their potential to reach an extraordinarily large number of tobacco users are extensively documented [7]. Quitlines serve all 50 states as well as Puerto Rico, Guam, and the District of Columbia [19]. Nevertheless, Quitlines are grossly underutilized, reaching only about 1–2% of all smokers annually [6], and even modest increases in their reach could impact tobacco use prevalence at the population level [8, 20].

### Tobacco cessation and primary care

Primary care provides an ideal setting to address tobacco cessation because there is an established relationship and coordination of care, tobacco use can be routinely assessed, and 80% of all tobacco users see a physician at least annually [21]. Federally Qualified Health Centers and Community Health Centers (referred to collectively as CHCs) are extraordinary venues for reaching low SES populations with evidence-based treatment for tobacco cessation [7, 22]. In 2018, CHCs provided primary care to over 28 million patients, ~ 40% were minorities and ~ 70% had incomes below the federal poverty level [23]. Recommendations from the Centers for Disease Control and Prevention and others include aggressive, targeted, proactive strategies to increase the utilization of Quitlines including formal partnerships with primary care [4, 5, 9].

Electronic health record (EHR)-based strategies at the point of care can dramatically increase the reach of Quitline treatment, are feasible, and can fit within existing clinical systems [24, 25]. Ask–Advise–Connect (AAC; also called “eReferral”) utilizes widely available, standards-based EHR capabilities to systematically assess the tobacco use status of patients, advise them to quit, and directly and electronically connect interested tobacco users with evidence-based Quitline treatment. Prior research has clearly documented the efficacy of AAC (a 13-to-30-fold increase in the proportion of tobacco users who receive evidence-based treatment from a Quitline compared to the previously recommended standard of care) [24, 25], yet it has not been widely adopted and implemented in primary care settings. Additionally, there is a potential for increasing the effectiveness of AAC by combining it with other approaches (text messaging, counseling).

Text messaging is effective for improving tobacco cessation outcomes and engagement with treatment [26–28]. Text messaging may be a scalable approach for increasing the reach of evidence-based treatment for tobacco use given that mobile phones and text messaging are widely used even in low SES populations [29], are very low cost, can be automated, and can potentially reach a high proportion of tobacco users [28].

Proactive counseling is another effective strategy for improving engagement with tobacco cessation treatment [30–32] because it helps patients overcome barriers to accessing and adhering to tobacco cessation interventions. It is highly feasible in primary care given increasing integration with behavioral health and the widespread use of health educators and patient navigators [33].

### Multilevel implementation strategies to improve Quitline engagement

Multilevel implementation strategies (MLIs) target change in more than one contextual level (e.g., healthcare system, provider, patient) to influence health behavior, health care practice change, and health outcomes [34–37]. Although multiple national organizations have underscored the need to implement MLIs [38–40], there are few data addressing how and when to implement MLIs for tobacco cessation in primary care or how interventions at multiple levels interact and influence each other.

### Adaptive interventions

Adaptive interventions can be used to operationalize the sequencing and integration of MLIs. An adaptive intervention is an intervention design involving a sequence of intervention decisions that are individualized such that ongoing information from the individual (e.g., patient) or organization (e.g., clinic) is used to guide whether and how to intervene over time [41–43]. Modifying intensity or type of strategy over time can improve outcomes if an individual or organization is not responding or may reduce costs and burden if more resource intensive strategies are not necessary [43]. Adaptive stepped care interventions [42–45] have the potential to conserve scarce resources (e.g., counseling) for the patients that need them most and minimize unnecessary treatment burden. The Sequential Multiple Assignment Randomized Trial (SMART) is an experimental design developed explicitly for the purpose of optimizing adaptive interventions [46–48].

### Aims and objectives

The overall objective of this research, entitled “QuitSMART Utah,” is to increase the Reach and Impact of evidence-based tobacco cessation treatment in order to reduce the prevalence of tobacco use among low SES populations. The scientific premise of the study is based on (1) substantial consensus that evidence-based tobacco cessation treatments delivered via Quitlines are effective but grossly underutilized [4–7, 9], (2) data demonstrating that EHR based strategies at the point of care can dramatically increase the reach of Quitline treatment, are feasible, rely on ubiquitous EHR capabilities, and can fit within existing clinical workflows [24, 25], (3) results demonstrating that technology-based approaches such as text messaging can increase the reach of evidence-based treatment [26], and (4) evidence highlighting the effectiveness of proactive counseling in increasing the reach of and impact of treatment [26, 30–32].

The aims of QuitSMART Utah are threefold: (1) evaluate healthcare system, provider, and patient level implementation strategies to increase the Reach of evidence-based treatment for tobacco use (delivered via Quitline). Reach is defined as the proportion of smokers who enroll in Quitline delivered treatment. (2) Evaluate healthcare system, provider, and patient-level implementation strategies to increase the Impact of Quitline treatment. Impact is defined as Reach × Efficacy, where efficacy is defined as the proportion of tobacco users who enroll in Quitline delivered treatment that successfully quit. (3) Evaluate characteristics of healthcare system, providers, and patients that may influence tobacco use outcomes.

## Methods

We will conduct a pragmatic, multilevel, Sequential Multiple Assignment Randomized Trial (SMART) in CHC primary care clinics across Utah. The protocol for the study has been reviewed and approved by the University of Utah Institutional Review Boards (IRBs). The project is registered at clinicaltrials.gov (NCT03900767).

### Conceptual framework

**Fig. 1:**
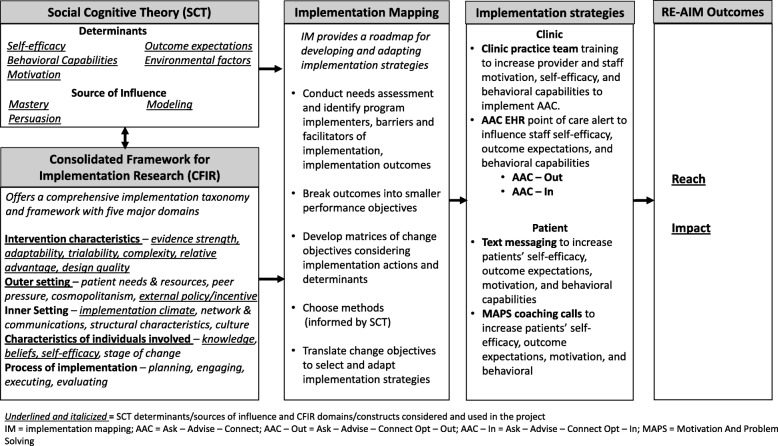
Conceptual framework

The conceptual framework illustrates how various frameworks and models inform the development or adaptation of components of the MLIs and describe potential mediators, moderators, and outcomes (Fig. 1). We use Implementation Mapping (IM) [49–51] to organize our conceptual framework. IM is a systematic process for planning implementation strategies. It is based on Intervention Mapping, a planning framework that has been used extensively in the development and implementation of multilevel interventions. Our conceptual framework includes RE-AIM [52], Social Cognitive Theory (SCT) [53, 54], and the Consolidated Framework for Implementation Research (CFIR) [55]. RE-AIM [52] defines our primary outcomes of Reach and Impact (Reach × Efficacy). SCT identifies the determinants of behavior (e.g., motivation, self-efficacy, behavioral capability) and methods for influencing these determinants at multiple levels (e.g., patient, clinic) [53, 54]. CFIR includes a comprehensive, multi-level taxonomy of factors that influence implementation (e.g., inner and outer settings of the organization, implementation processes). SCT and CFIR are synergistic in that SCT specifies the core determinants and predictive relations driving implementation behaviors, while CFIR guides the consideration of what implementation behaviors are important to target and describes contextual factors that can influence implementation.

In designing the MLIs, we identified SCT determinants (e.g., self-efficacy, motivation, behavioral capabilities) among primary care providers (PCPs), staff, and patients in four of the five CFIR domains (i.e., strategy characteristics, inner setting, outer setting, characteristics of involved individuals) and chose strategies that addressed these factors (Fig. 1). All implementation strategies will be adapted in collaboration with potential adopters and implementers.

### Setting

Thirty-three primary care clinics within 11 CHC organizations will be recruited to participate in the project. All CHC organizations and clinics will be recruited in partnership with the Association for Utah Community Health (AUCH), the federally designated Primary Care Association that represents Utah’s 13 CHC organizations. The 11 participating CHCs serve ~ 88,000 unique adult patients annually with a mix of patient demographics, including > 50% minority (48% Latino; 9% American Indian), 52% uninsured, and 68% living under the federal poverty level [56]. CHCs range in size from one to seven primary care clinics per organization and include both rural and urban clinics.

### Participants

#### Participants

Participants will be 6000 current tobacco users ≥ 18 years of age who speak English or Spanish, have a working cellphone that can accept texts and calls, and who present at participating clinics. Patients will be identified as tobacco users during the clinical encounter. Tobacco use includes cigarettes; little cigars, cigars, or cigarillos; vape pen, Juul, or e-cigarettes; and smokeless tobacco/snus.

#### Organization and clinic staff

All organization and clinic staff of participating CHCs and clinics will be eligible to participate.

### Research–practice partnership

Community and stakeholder engagement is a basic premise of patient-centered outcomes research, an integral component of our conceptual framework, and fundamental for the Implementation Mapping process. The project is being conducted in partnership with AUCH, CHCs, the Utah Department of Health (UDOH) Tobacco Prevention and Control Program (TPCP), and the investigator team. The investigator team, UDOH, and AUCH jointly identified tobacco control as a priority among CHCs in Utah, specifically, the need to integrate e-referral between clinics and the Utah Tobacco Quit Line (UTQL). UDOH provides UTQL treatment free of cost to all residents of Utah and has partnered with the investigator team to facilitate data sharing between the UTQL, UDOH, and the investigator team.

### Evidence-based practice to be implemented: Utah Tobacco Quit Line treatment

Patients who enroll in UTQL-delivered treatment are eligible to receive free behavioral treatment via phone counseling, e-chat, online, or text platforms, as well as nicotine replacement therapy (NRT) if there are no medical contraindications.

### Clinic implementation strategy

Participating clinics will be assigned to either an opt-in (AAC-In) or an opt-out version of AAC (AAC-Out). The AAC EHR configuration will be developed by the research team in conjunction with CHC partners. Both AAC-In and AAC-Out are based on EHR capabilities that are required for EHR certification in the US, including tobacco-use documentation and the ability to securely send electronic referrals with a summary of the patient’s health record automatically added as an attachment.

#### AAC-Opt In

To date, procedures for increasing tobacco cessation treatment uptake in primary care, including AAC, have generally utilized opt-in policies in which the EHR provides the option for clinical staff to initiate advice to tobacco users to quit and to offer an automated, electronic connection to the Quitline.

#### AAC-Opt Out

AAC-Opt Out is based on work in behavioral economics on “nudges,” which refer to “any aspect of the choice architecture that alters people’s behavior in a predictable way without forbidding any options or significantly changing their economic incentives” [57]. Setting up the default option in a specific way constitutes a nudge and can have extremely potent effects [58]. In the opt-out approach, the EHR requires clinical staff to advise tobacco users to quit and offer a connection to the Quitline prior to moving forward or to opt-out. Opting out is simple and generally requires only a single keystroke, consistent with nudge choice architecture providing an easy way to avoid the intervention.

### Clinic staff implementation strategy

#### Training

Prior to AAC implementation, the clinic practice team at each clinic will receive a brief (e.g., 30 min), in-person training designed to increase provider and staff motivation, self-efficacy, and behavioral capabilities to implement AAC. The trainings will be delivered by an employee of AUCH.

### Patient facing implementation strategies

We define patient facing implementation strategies as those designed to increase patient engagement with the evidence-based treatment (i.e., UTQL).

#### Text messaging

Patients will receive Health Insurance Portability and Accountability Act (HIPAA)-compliant text messages that offer repeated opportunities to enroll in UTQL treatment with a simple two-touch response [26].

#### MAPS

Patients will receive brief counseling calls by health coaches trained in Motivation And Problem Solving (MAPS) [44–46]. MAPS is designed to help patients address the larger context in which tobacco use occurs, motivate patients towards a long-term goal of quitting tobacco, and identify and address barriers to quitting and engaging in treatment. MAPS has demonstrated effectiveness for addressing the entire cessation process of considering quitting, cessation, and Quitline treatment engagement [30–32, 60].

### Study design

**Fig. 2:**
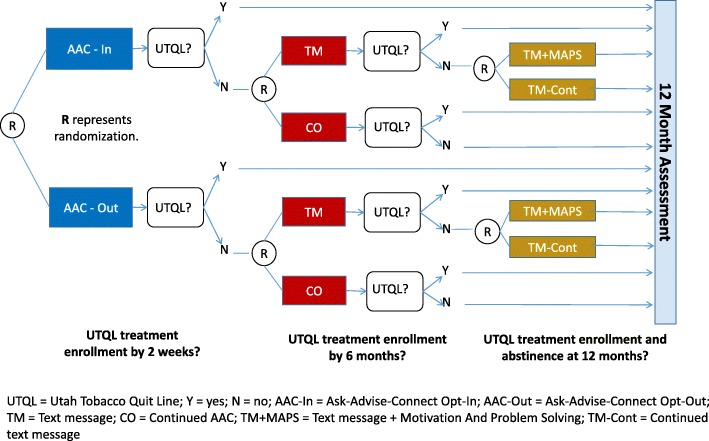
Study design

**Fig. 3:**
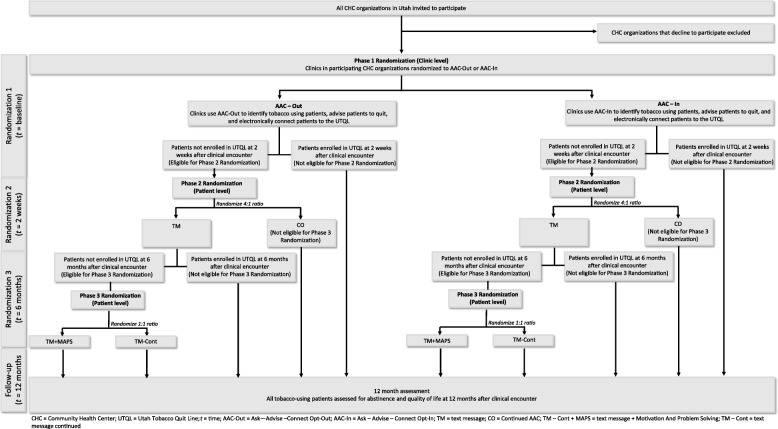
Clinic and patient flow diagram

QuitSMART Utah is a multilevel, three-phase, Sequential Multiple Assignment Randomized Trial (SMART), conducted in 33 CHC clinics within 11 CHC organizations with 6000 tobacco users (Fig. 2). Descriptions of the clinic and patient conditions are listed in Table 1. See Fig. 3 for the clinic and patient flow diagram.

**Table 1:** Description of clinic and patient conditions

Randomization (level)	Condition	Description
Phase 1 (clinic)		
	Ask–Advise–Connect Opt-In (AAC-In)	AAC-In consists of an EHR based point of care reminder that allows medical staff to choose when to perform Advise and Connect (i.e., the default does not require an action).
	Ask–Advise–Connect Opt-Out (AAC-Out)	AAC-Out consists of an EHR based point of care alert that requires clinic staff to Advise and Connect tobacco users to the UTQL or to “opt out” (i.e., the default requires an action; Advise and Connect or Opt Out).
Phase 2 (patient)		
	Text message (TM)	Patients will receive text messages with a two-touch response that directly connects patients to the UTQL. Texts will be sent once per week for the first month and once per month for the remaining 5 months (10 texts total in 6 months).
	Continued AAC (CO)	Continued EHR intervention (i.e., AAC-In or AAC-Out). Clinic staff will perform AAC if patient returns to clinic.
Phase 3 (patient)		
	Continued text message (TM-Cont)	Patients will continue to receive text messages with a two-touch response that directly connects patients to the UTQL. Texts will be sent once per month for 6 months.
	Text message + MAPS (TM + MAPS)	Patients will continue to receive text messages with a two-touch response that directly connects patients to the UTQL, plus two brief telephone calls from patient navigators/health educators trained in MAPS counseling.

*EHR* electronic health record, *UTQL* Utah Tobacco Quit Line, *MAPS* Motivation And Problem Solving

In the first phase, clinics will be randomized to either AAC-In or AAC-Out. After the initial clinical encounter with a patient, those patients who do not enroll in UTQL treatment in response to the AAC condition after ~ 2 weeks will be individually randomized to receive either text messaging (TM) or continued AAC only (CO) in a 4:1 ratio (4 to TM for every 1 to CO). Patients who enroll in the UTQL receive no further interventions. Six months after the clinical encounter, participants in the TM condition who are non-responders (i.e., have not enrolled in UTQL treatment) will be randomized to receive either continued text messages (TM-Cont) or text messages + MAPS (TM + MAPS) in a 1:1 ratio. Patients who enroll in UTQL receive no further interventions. All randomization sequences will be generated using random permuted blocks with random block sizes to assure balanced randomization over time. Clinic randomization will be stratified by CHC and clinic size, with several of the smaller CHCs grouped into the same stratum to provide sufficient clinics for stratification by clinic size within this stratum. Patient-level randomization will be stratified by clinic and by the treatment groups of earlier phases in the SMART design.

### Stakeholder and patient engagement in the development/adaptation of implementation strategies

To ensure implementation strategies align with the contexts in which they are delivered, the implementation protocols and materials will be developed using the IM process in partnership with the implementers of the study components using the following activities: (1) meetings with CHC organization adopters and implementers, (2) patient and study advisory committees, and (3) clinic workflow assessments and EHR adaptation usability assessments.

The research team will conduct semi-structured interviews with potential adopters and implementers at the CHCs. The interviews will include questions regarding successes and barriers for ongoing and past tobacco control efforts, goals and expectations for the current study, and other constructs expected to influence successful implementation. As we better understand implementation behaviors and determinants through discussion and observation of clinical contexts, we will present these to our stakeholders for validation and/or revision. Patient and study advisory committees, including representation from patients, each of the participating CHCs, UDOH, AUCH, and the investigator team, will meet throughout the duration of the project to inform development of all implementation strategies and to give input on study procedures, outcome assessments, and study dissemination.

Consistent with sociotechnical principles of design [61] and implementation science frameworks [55], HIT implementation strategies such as AAC should be designed to fit within the context of the existing clinical system workflow to avoid disruption [59] without compromising the active ingredients of the strategy. The research team will perform mixed methods clinic workflow evaluations in clinics prior to designing AAC to (1) understand the needs and assets of the existing clinical workflow to minimize disruption and enhance AAC delivery and (2) identify possible implementation barriers to be addressed during the clinic practice team training. The research team will observe patient clinical encounters to collect data regarding who, what, when, where, and how (i.e., the “five rights” of clinical decision support) [62] patient tobacco use is collected, stored, and communicated, cessation advice is delivered, and patients are referred to tobacco cessation services. Observations will be supplemented by paper-based surveys administered to front office and clinical staff regarding their roles and responsibilities related to identification of tobacco users, recommendations to quit, and referral to cessation services. Following EHR adaptations (i.e., AAC-In, AAC-Out), we will conduct iterative usability testing with EHR screen “mock-ups” to evaluate fit within existing clinic workflows, obtain user feedback, and address barriers to AAC implementation.

### Outcomes measures and data collection

Outcomes and assessments are guided by the conceptual framework (Fig. 1) and represent either our outcomes or potential determinants/moderators of outcomes.

#### Patient

The primary outcome, Reach, is defined as the proportion of tobacco users who enroll in UTQL treatment. Patient enrollment in the UTQL (i.e., the numerator of Reach) will be assessed and obtained from the UTQL vendor on a continuous basis throughout the duration of the study. The total number of tobacco-using patients seen at participating clinics (i.e., the denominator of Reach) will be gathered from the EHR at participating clinics on a continuous basis. Use of tobacco/nicotine products (e.g., smokeless, cigars, e-cigarettes, vaporizers) and patient demographics (e.g., race/ethnicity, insurance status, primary language, age) will be collected via the EHR.

The other primary outcome, Impact, is defined as the product of Reach and Efficacy. Efficacy is defined as patient abstinence status at 12 months following the patients’ initial clinical encounter among patients who enrolled in the UTQL. Tobacco-using patients will be contacted to complete a self-report abstinence survey at 12 months following the initial clinical encounter. Patient self-reported abstinence status will be assessed using recommendations for cessation induction trials (i.e., 7- and 30-day point prevalence abstinence) [63]. A subset of patients (*n* = 300) self-reporting abstinence at 12 months will complete biochemical verification of abstinence (i.e., cotinine) using mailed saliva sample kits.

#### Clinic and organization

CHC system and clinic practice team implementation determinants and outcomes will be assessed via staff surveys, qualitative interviews, data from Uniform Data System (UDS) reporting (i.e., the standardized reporting system) [64], and the EHR. We will conduct in-depth interviews with clinic staff halfway through study completion that will be recorded and transcribed for analysis. We will conduct a thematic content analysis using iterative and deductive coding to better understand implementation in clinics and further inform dissemination efforts [65–68]. We will collect survey data on implementation determinants from staff from each clinic (e.g., medical assistants, nurses, doctors, clinic directors) at three time points (i.e., prior to AAC implementation, midway through study completion, at the conclusion of patient follow-up). Assessments will measure changes in implementation self-efficacy, outcome expectations, and behavioral capabilities (Fig. 1). We will assess selected constructs from key CFIR domains such as the inner setting, intervention characteristics, outer setting, characteristics of individuals, and process [55]. We will obtain data on clinic characteristics such as number of patients served each year, patient demographics, and health insurance status, from UDS. We will collect data regarding fidelity of the clinic practice team to administering AAC routinely from the EHR.

### Statistical analyses and power calculations

#### Analytic plan

The richness of the three-phase SMART design provides the opportunity to test multiple hypotheses [69, 70]. Our primary hypotheses address the capability of the specific interventions in each phase to extend Reach and Impact beyond the preceding phase: Is AAC- Out superior to AAC-In at 2 weeks? Is TM superior to CO at 6 months among those who fail to use the Quitline after the ACC interventions? Is TM + MAPS superior to TM-Cont at 12 months among those who fail to use the Quitline after both the AAC and TM interventions? Secondary analyses will compare embedded dynamic treatment regimens.

#### Primary analyses for reach

Our primary analyses of Reach will be performed using generalized estimating equations (GEE) to account for clustering of patients within clinic [71, 72]. We will use robust standard errors used for statistical inference, which will incorporate appropriate upward covariance adjustment to correct for the downward bias of these estimates when the number of clusters is limited [73]. Separate analyses will be applied for the different treatment comparisons at each stage of the three-phase design. At each phase, GEE will be applied to a binary outcome model and will include the CHC as a clinic-level factor and patient level covariates as predictor variables to control for baseline imbalances that can occur when clinic level randomization is used [74]. The GEE analyses for the phase 2 and phase 3 treatment comparisons will also include indicator variables for the patients’ randomized assignments at previous phases as covariates.

Our primary analyses will estimate (1) the effect of the AAC-Out vs. AAC-In on Reach at 2 weeks for the full cohort of all randomized patients, (2) the effect of TM vs. CO on 6-month Reach in the subcohort without UTQL use by week 2, and (3) the effect of the TM + MAPS vs. TM on month 12 Reach in the subcohort with no UTQL use by month. Our primary phase 2 and phase 3 comparisons will average over patients exposed to different interventions in the previous phases. Secondary contrasts will compare the effects of the phase 2 and phase 3 interventions within subgroups exposed to specific interventions in the earlier phases.

#### Primary analyses for impact

GEE analyses with a binary outcome model will also be used for Impact, defined by a binary indicator which is equal to 1 only for patients who both use the UTQL by a designated follow-up time and are abstinent at month 12. We will also evaluate efficacy by conducting secondary analyses to compare 12 month abstinence among patients assigned to the respective treatments (or treatment regimens) within the subgroups who use the UTQL by the designated follow-up times. For example, the effect of AAC-Out vs. AAC-In on efficacy will be evaluated by comparing the proportions of patients who report 12 month abstinence between those in the AAC-Out and AAC-In groups who use the UTQL by 2 weeks.

#### Multiple comparisons

Evaluating multiple hypotheses under the SMART design may inflate studywise Type 1 error. We address this issue by designating Reach and Impact as co-primary outcomes and testing focused primary hypotheses concerning the effects of the three interventions at the individual stages of the design. Other analyses are secondary or exploratory. Because our hypothesis tests for the three interventions address distinct questions, we will apply a Bonferroni-Holm multiple comparison adjustment to account for the two co-primary outcomes (Reach and Impact) when evaluating each intervention, but will not perform additional multiple-comparison adjustment for the three interventions [75].

#### Statistical power

The power calculations for the AAC-Out vs. AAC-In comparison assume an intraclass correlation for each outcome of 0.02 and a coefficient of variation of the clinic sizes of 0.88 based on a census of the 33 participating clinics. The calculations for TM vs. CO in phase 2 assume ≥ 80% of subjects fail to use the UTQL by week 2, and the calculations for TM + MAPS vs. TM-Cont in phase 3 assume both that ≥ 80% of subjects fail to use the UTQL by week 2, and ≥ 80% of those assigned to TM in phase 2 fail to use the UTQL by month 6. Under these assumptions, the 6000-patient SMART design will provide 80% power (two-sided α = 0.025 to account for two co-primary outcomes) to detect increases in Reach of 6.2%, 2.8%, and 3.6% from assumed control group percentages of 10%, 5%, and 10%, for the AAC-Out vs. AAC-In, TM vs. CO, and TM + MAPS vs. TM-Cont. comparisons, respectively. The corresponding minimum detectable effects for Impact are 3.5%, 1.5%, and 1.9%, relative to assumed control group percentages of 2.0%, 1.0%, and 2.0%, respectively.

## Discussion

QuitSMART Utah is a pragmatic, cluster-randomized clinical trial with the objective of increasing the Reach and Impact of evidence-based tobacco cessation treatment among low SES populations. The innovative SMART design will provide key guidance on which strategies are most effective for implementing the linkage between primary care and Quitlines, as well as whether progressively more resource intensive strategies provide unique, incremental effectiveness over and above the implementation of less resource intensive approaches. QuitSMART Utah will provide critical data regarding the impact of pragmatic and scalable strategies at both the clinic and patient level and advance the field of implementation science by testing key constructs hypothesized to influence implementation effectiveness. The ultimate goal is to decrease tobacco use at the population level and reduce the disproportionate burden of tobacco related morbidity and mortality among low SES populations.

There are several operational and practical challenges to conducting QuitSMART Utah including (1) EHR technical capabilities that can impact the ability to both implement the AAC conditions exactly as envisioned and to randomize individual clinic sites to study conditions in the first step of the SMART design, (2) CHC decisions to change EHR vendors during the project, (3) change in the UTQL vendor during the project, and (4) obtaining approval from multiple IRBs, including a Tribal IRB.

### EHR technical capabilities

During the first phase of the SMART design, clinics will be randomized to receive ether the opt-in or opt-out condition for implementing AAC. Adapting AAC for the wide variety of EHR vendors in a consistent way across different EHRs is challenging. For example, the investigative team has implemented AAC-In and AAC-Out in several healthcare systems using an EHR product that provides the technical capability to embed logic such that a “pop-up” alert window can be triggered after a patient is identified as a tobacco user. To get past the pop-up window, the clinical team must either provide advice and an offer of connection to evidence-based cessation treatment or “opt-out.” However, not all of the EHR products used by CHCs provide the capability to embed logic that can trigger these kinds of prompts/reminders. These limitations may impact the ability to implement AAC-In and AAC-Out precisely as conceptualized, and the team is actively attempting to find “work-arounds.”

The study includes 11 CHCs and 33 individual clinics. Although all of the CHCs agreed to allow individual clinic sites to be randomized to AAC study condition, technical constrains with at least one of the EHRs may make this challenging. For those CHCs, the alternative would be to randomize the CHC systems, rather than clinics, to AAC-In or AAC-Out. This would reduce the study power for the phase 1 randomization, and we are actively looking for solutions to this challenge. The clinic assignment will not affect power to detect differences for the comparisons in the second and third phases since we will randomize individuals within clinics. Additionally, we have recruited more CHCs and clinics than were included in our original sample size calculation. As such, power for detecting differences in the AAC-Out vs. AAC-In condition may be relatively unaffected.

### Change in EHR vendor

Another issue, also related to the EHR, is that one of the CHCs decided to change their EHR product after study initiation. Since the new EHR product will be implemented after the scheduled trial kick-off, this CHC will need to join the trial later than other CHCs. Nevertheless, because the CHC is changing to one of the three EHRs that are already part of the study, we will have experience modifying that EHR platform.

### Change in UTQL vendor

The goal of the implementation strategies evaluated in QuitSMART Utah center around connecting tobacco users to evidence-based tobacco cessation treatment, with the most commonly implemented treatment being Quitlines [19]. One practical challenge encountered in our study is that the UTQL changed Quitline vendors midway through the first year of QuitSMART Utah. We had already begun working with the original vendor, including negotiating costs, how to implement bi-directional electronic referrals via AAC, obtaining data on patient enrollment by clinic, and other procedural issues related to data transfer. Hence, we needed to begin these discussions a new with the new vendor once the change was implemented.

### Complex data collection and reporting procedures with multiple IRBs

We have worked closely with our study partners and IRBs to develop a process for collecting outcome data that capitalizes on data sources that our partners (i.e., AUCH and UDOH) already utilize. Although the process we developed will minimize patient and clinic practice team burden, the procedures include extensive data sharing between partner organizations and have resulted in the need for > 10 data sharing agreements and approval from three separate IRBs. Gaining approval from multiple IRBs from distinct entities (i.e., academic, department of health, tribal) can present a number of challenges, as the entities may have conflicting procedural requirements. For example, two of the three IRBs have policies that a study must be approved by other IRBs before their board can approve the study (i.e., the university IRB requires tribal IRB approval prior to reviewing the project; the tribal IRB requires university approval prior to reviewing the project). To resolve this and other similar issues, we have held numerous meetings with the three IRBs and facilitated communications between the IRB directors.

Review boards representing communities that have experienced injustice from scientific research, such as American Indian populations, often include additional policies and protocols to protect the rights of their communities. Two of the CHC organizations participating in this project serve primarily American Indian populations from two different tribes, one of which has an independent Tribal IRB. Approval from the Tribal IRB is a time-intensive process, designed to build relationships between the researchers and the communities with whom the research is being conducted to ensure the rights of the community are protected. We have spent over 1 year in this process and have traveled over 4000 miles to gain the necessary support to present our application at the Tribal IRB.

## Summary

QuitSMART Utah will be conducted in real-world practice settings that reach underserved populations. There is minimal disruption to clinical workflow to maximize external validity and the potential for broad population-level scale up. Some of the challenges mentioned above will likely be relevant in future real-life dissemination of the implementation strategies developed and tested here. Thus, thorough documentation of these issues and the solutions applied to overcome them is critical. Our team will assess and describe these issues as well as any others to provide guidance for future implementation and dissemination efforts. Eventual broad-scale dissemination of the results and implementation strategies developed in QuitSMART Utah could increase tobacco cessation at the population level and reduce the disproportionate burden of tobacco-related morbidity and mortality among low SES populations.

### Update to study design and statistical analysis

In the time from submission of this manuscript to acceptance, there has been a change in the study protocol. First, two clinics within a single CHC organization combined to form one clinic. Therefore, our total sample of clinics will change from 33 clinics to 32 clinics. Second, the study team was unable to develop an ‘Opt-Out’ version of AAC in one of the EHR systems due to technical limitations (as described in the discussion). Therefore, the Phase 1 randomization will occur only among the CHC clinics that use the two EHR systems that can be configured to both AAC-In and AAC-Out (*n* = 21 clinics). Clinics that use the third, nonconfigurable EHR will not be randomized in phase 1 (*n* = 11 clinics), and will all receive the EHR intervention of AAC-In. Patients in clinics using the nonconfigurable EHR will be randomized in the patient level randomizations (Phase 2 and Phase 3). Our primary analyses of the AAC-Out vs AAC-In comparison will be restricted to the 21 clinics with EHRs that allow them to be randomized to either condition, and will thus exclude the clinics that use the EHR that cannot be configured to AAC-Out. Comparisons of TM vs. CO and of TM+MAPS vs. TM will be performed for patients who receive care in all 32 clinics. The updated power calculations for the AAC-Out vs. AAC-In comparison continue to assume an intraclass correlation for each outcome of 0.02, and now assume a coefficient of variation of the clinic sizes of 0.96 based on a census of the 21 participating clinics that use the two configurable EHRs. The 21 clinics have a projected total of 4,019 patients out of the projected total of 6,000 patients across all 32 participating clinics. Our updated power analyses indicate that the minimum detectable effect for Reach of the AAC-Out vs. AAC-In comparison is an 8.1 percentage point increase in AAC-Out relative to an estimated reach of 10% in AAC-In. The corresponding minimum detectable effect for Impact is 4.8 percentage points in AAC-Out relative to an assumed Impact of 2.0% in AAC-In. In addition to these changes in the design and power calculations, we have also modified our plans for addressing missing abstinence data in the computation of the Impact Outcome. We now plan to apply multiple imputation in cases where patients who fail to respond to the abstinence questionnaire.

## Data Availability

Not applicable.

## Abbreviations

AAC: Ask–Advise–Connect
AUCH: Association for Utah Community Health
CFIR: Consolidated Framework for Implementation Research
CHCs: Federally Qualified Health Centers/Community Health Centers
EHR: Electronic health record
HIPAA: Health Insurance Portability and Accountability Act
IM: Implementation Mapping
IRB: Institutional Review Board
NRT: Nicotine Replacement Therapy
SCT: Social Cognitive Theory
SES: Socioeconomic status
SMART: Sequential Multiple Assignment Randomized Trial
TPCP: Tobacco Prevention and Control Program
US: United States
UDOH: Utah Department of Health
UTQL: Utah Tobacco Quit Line
MAPS: Motivation And Problem Solving
TM: Text messaging
CO: Continued
TM-Cont: Continued text messaging
TM + MAPS: Text messaging + MAPS
HIT: Health Information Technology
SF-12: 12-Item Short Form Survey
UDS: Uniform Data System
